# Mesenchymal stem cells provide prophylaxis against acute graft-versus-host disease following allogeneic hematopoietic stem cell transplantation: A meta-analysis of animal models

**DOI:** 10.18632/oncotarget.11238

**Published:** 2016-08-12

**Authors:** Li Wang, Haiyan Zhang, Lixun Guan, Shasha Zhao, Zhenyang Gu, Huaping Wei, Zhe Gao, Feiyan Wang, Nan Yang, Lan Luo, Yonghui Li, Lili Wang, Daihong Liu, Chunji Gao

**Affiliations:** ^1^ Department of Hematology, Chinese People's Liberation Army (PLA) General Hospital, Beijing, China; ^2^ Department of Hematology and Oncology, Laoshan Branch, No. 401 Hospital of Chinese PLA, Qingdao, China; ^3^ Department of Hematology, Linyi People's Hospital, Linyi, China

**Keywords:** mesenchymal stem cells, graft-versus-host disease, hematopoietic stem cell transplantation, meta-analysis, animal experimentation

## Abstract

A meta-analysis of animal models was conducted to evaluate the prophylactic effects of mesenchymal stem cells (MSCs) on acute graft-versus-host disease (aGVHD) after allogeneic hematopoietic stem cell transplantation. A total of 50 studies involving 1848 animals were included. The pooled results showed that MSCs significantly reduced aGVHD-associated mortality (risk ratio = 0.70, 95% confidence interval 0.62 to 0.79, *P* = 2.73×10^−9^) and clinical scores (standardized mean difference = −3.60, 95% confidence interval −4.43 to −2.76, *P* = 3.61×10^−17^). In addition, MSCs conferred robust favorable prophylactic effects on aGVHD across recipient species, MSC doses, and administration times, but not MSC sources. Our meta-analysis showed that MSCs significantly prevented mortality and alleviated the clinical manifestations of aGVHD in animal models. These data support further clinical trials aimed at evaluating the efficacy of using MSCs to prevent aGVHD.

## INTRODUCTION

Allogeneic hematopoietic stem cell transplantation (allo-HSCT) is the only curative modality for many hematological malignancies. The number of patients undergoing this procedure is rapidly increasing because of the development of novel allo-HSCT strategies and improved supportive treatments [1]. However, life-threatening complications, especially acute graft-versus-host disease (aGVHD), are frequently encountered after allo-HSCT and can limit the widespread use and success of this important therapy [1, 2]. Currently, a variety of prophylactic strategies, including T-cell depletion and immunosuppressive agents, are used to prevent aGVHD. However, T-cell depletion impairs the graft-versus-leukemia effect and has been associated with an increased rate of primary disease relapse [1]. Furthermore, pharmacological strategies are associated with impaired immune reconstitution in recipients [3]. Therefore, novel prophylactic strategies for aGVHD are urgently needed.

Mesenchymal stem cells (MSCs) are considered ideal candidates for cell therapy during allo-HSCT because of their unique immunomodulatory and reparative properties [4]. MSCs are currently generating significant interest because they confer potential prophylactic effects against aGVHD following allo-HSCT [5, 6], and several relevant randomized controlled trials (RCTs) have been published [7–10]. However, the results of these studies have been inconsistent. Ning et al. [7] reported that the rate of grade II-IV aGVHD was significantly lower in an MSC-infused group than in the control group (11.1% versus 53.3%, respectively), whereas Liu et al. [8] reported that a higher rate of aGVHD was observed when participants received infusions of MSCs (51.8% versus 38.9% compared to recipients who did not receive MSCs). Because these studies included small sample sizes and their results were conflicting, our and other groups have conducted meta-analyses of relevant clinical trials, but the results have not indicated that the adoptive transplantation of MSCs prevents aGVHD [11, 12].

MSCs have been extensively studied in animal models as a prophylactic strategy against aGVHD after allo-HSCT. Similar to clinical trials, studies using animal models have produced conflicting results. Here, we perform the first meta-analysis of these animal models to provide recommendations for designing future clinical trials.

## RESULTS

### Study selection and characteristics

We identified a total of 2305 potentially relevant studies. After removing duplicates and screening article titles and abstracts, 2167 non-relevant studies were excluded. The full texts of the remaining 138 studies were screened. This led to the exclusion of an additional 88 studies that did not meet the eligibility criteria. The excluded full-text studies and the reasons for their exclusion are listed in Supplementary Table 1. Finally, 50 studies involving 1848 animals (1067 MSC recipients and 781 controls) and 93 and 41 comparisons that assessed aGVHD mortality and clinical scores, respectively, were included in the meta-analysis (Supplementary Figure 1) [13–62]. The majority of the studies used a previously described clinical scoring system [63] to assess the severity of aGVHD (a higher clinical score indicates more severe aGVHD). The characteristics of the included studies are listed in Tables 1 and Supplementary Table 2.

**Table 1:** Characteristics of the included studies

Characteristics	No. of comparisons
No. of publications	50
No. of MSC arms	94
**Species receiving MSCs**	
Rat	7
Mouse	87
**MSC sources**	
Rat BM	7
Mouse BM	48
Human BM	13
Human UCB	9
Human UC	4
Mouse adipose tissue	3
Human adipose tissue	2
Human menstrual blood	1
Mouse skin	1
Human decidua	4
Human placenta	2
**Range of MSC doses**	0.02 × 10^6^ to 20 × 10^6^
**MSC administration time**	
Co-transplantation with allo-HSCT	46
Multiple doses including co-transplantation	21
Single or multiple doses, 1 day post-allo-HSCT	27

Abbreviations: BM, bone marrow; UCB, umbilical cord blood; UC, umbilical cord.

### Methodological quality evaluation

Five studies reported that animals were randomly assigned to an MSC or control group [13, 19, 33, 38, 44], and four studies indicated that the assessors were blinded to outcomes [15, 45, 53, 58]. The majority of the included studies reported compliance with animal welfare requirements and conflict of interest statements. However, none of the included studies mentioned allocation concealment or sample size calculations (Tables 2 and Supplementary Table 3).

**Table 2:** Methodological quality of the included studies

Quality score criterion	Proportion of studies (%)
Published in peer-reviewed journal	100
Randomization	10
Allocation concealment	0
Blinding of outcome assessors	8
Estimation of sample sizes	0
Compliance with animal welfare requirement	82
Conflict of interest statement	64

### Meta-analysis

**Figure 1:**
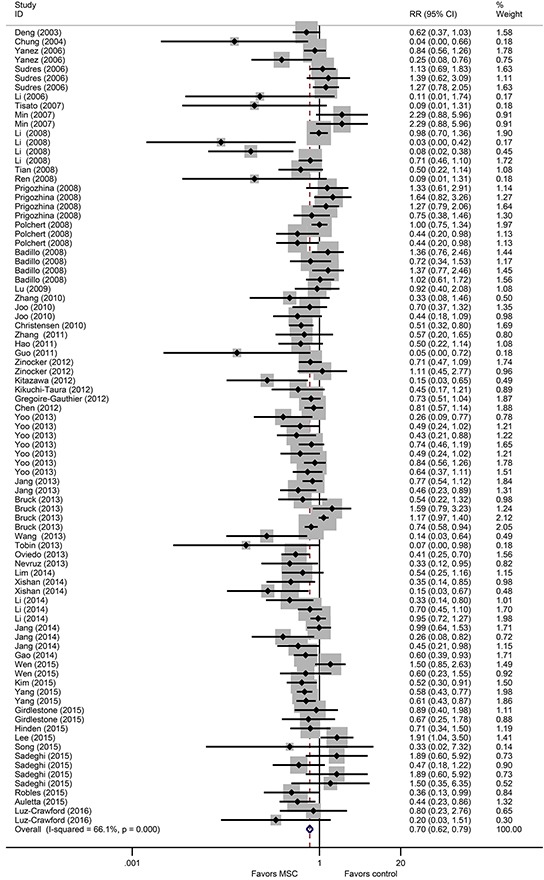
The prophylactic effect of MSCs on aGVHD mortality following allo-HSCT MSCs: mesenchymal stem cells, aGVHD: acute graft-versus-host disease, allo-HSCT: allogeneic hematopoietic stem cell transplantation, RR: risk ratio, CI: confidence interval.

**Figure 2:**
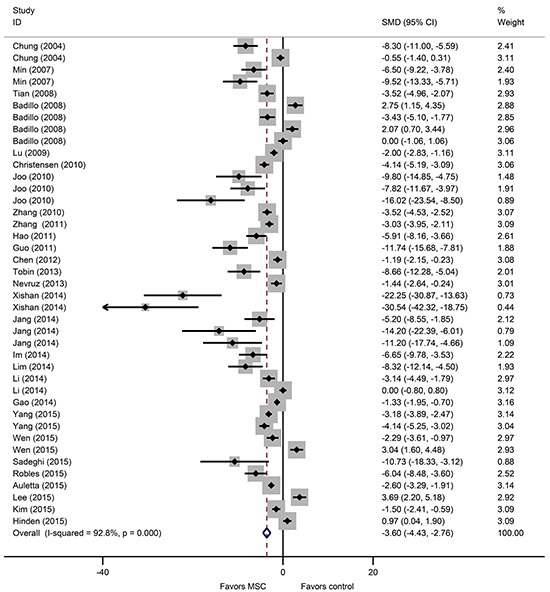
The prophylactic effect of MSCs on aGVHD clinical scores following allo-HSCT MSCs: mesenchymal stem cells, aGVHD: acute graft-versus-host disease, allo-HSCT: allogeneic hematopoietic stem cell transplantation, SMD: standardized mean difference, CI: confidence interval.

A total of 49 studies involving 93 comparisons examined the effect of MSCs on aGVHD-associated mortality in animal models of allo-HSCT [13–48, 50–62]. The pooled results indicated that aGVHD-associated mortality was significantly lower in the MSC groups than in the control groups (RR = 0.70, 95% CI 0.62 to 0.79, *P* = 2.73×10^−9^) (Figure 1). There was significant heterogeneity among the studies (I^2^ = 66.1%, *P* = 2.12×10^−18^) (Figure 1). In addition, 29 studies involving 41 comparisons examined the effect of MSCs on aGVHD-associated clinical scores [14, 15, 17, 19, 20, 22, 24, 27, 28, 33, 34, 36–39, 42, 44, 46, 47, 49–52, 55, 57–59, 61, 62]. The pooled analysis indicated that aGVHD-associated clinical scores were significantly lower in the MSC groups than in the control groups (SMD = −3.60, 95% CI −4.43 to −2.76, *P* = 3.61×10^−17^) (Figure 2). There was significant heterogeneity among the studies (I^2^ = 92.8%, *P* = 2.26×10^−92^) (Figure 2).

### Subgroup meta-analysis and meta-regression

Because there was significant heterogeneity among the studies, we conducted a subgroup meta-analysis using the following factors: recipient species, MSC source, MSC dose and administration time. We included only variables for which more than two comparisons were made. The subgroup meta-analysis demonstrated that MSCs provided similar beneficial prophylactic effects on the mortality and severity of aGVHD based on the recipient species, MSC dose and administration time (Supplementary Tables 4 and 5). In the MSC source data, the rate of aGVHD-associated mortality was significantly lower in groups administered mouse bone marrow (BM)-, human BM- and human umbilical cord blood (UCB)-derived MSCs than in the control groups (RR = 0.77, 95% CI 0.65 to 0.91; RR = 0.68, 95% CI 0.51 to 0.93; RR = 0.56, 95% CI 0.37 to 0.85, respectively) (Supplementary Table 4). However, there were no significant group differences when adipose tissue- and umbilical cord (UC)-derived MSCs were compared to the control group (RR = 0.49, 95% CI 0.23 to 1.06; RR = 0.51, 95% CI 0.20 to 1.31, respectively) (Supplementary Table 4). Consistent with the aGVHD mortality results, aGVHD clinical scores were significantly lower in the groups that received mice BM-, human BM-, and human UCB-derived MSCs than in the control group, and there was no significant difference between the human adipose tissue-derived MSC group and the control group (Supplementary Table 5).

To identify the potential source of heterogeneity, we conducted a meta-regression based on the factors mentioned above. The results indicated that the MSC source and dose accounted for a significant proportion of the heterogeneity in aGVHD-associated mortality (adjusted R^2^ = 5.41% and 1.73%, respectively) (Supplementary Table 4).

### Publication bias

**Figure 3:**
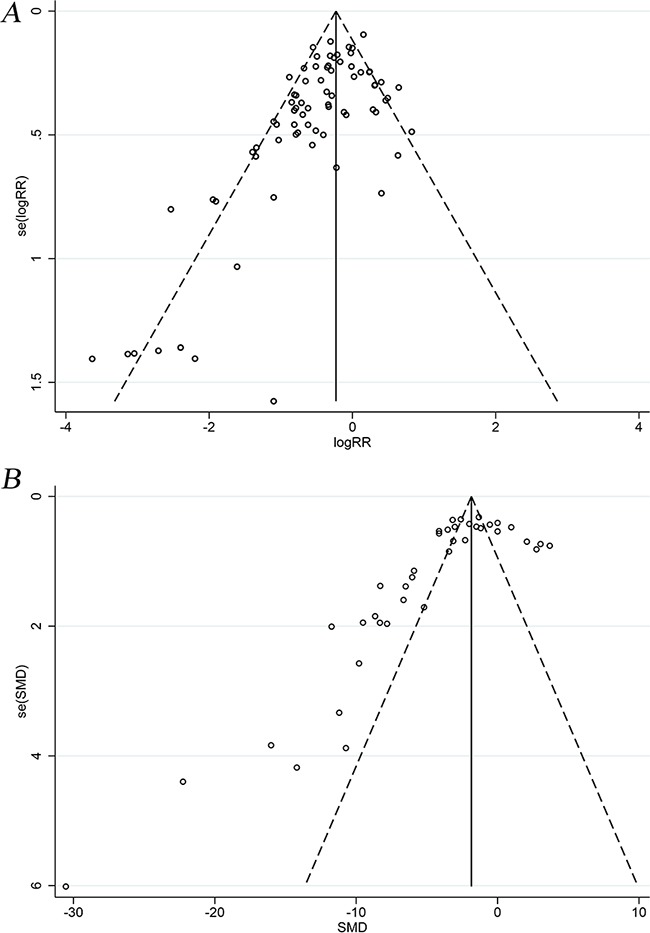
Funnel plots of aGVHD mortality and clinical scores **A.** Funnel plot of aGVHD mortality. **B.** Funnel plot of aGVHD clinical scores. aGVHD: acute graft-versus-host disease.

Funnel plots based on both aGVHD mortality and clinical scores showed asymmetry, suggesting the presence of publication bias (Figure 3). A subsequent Egger's test confirmed the existence of publication bias (*P* = 4.07×10^−6^, *P* = 0.001, respectively).

Small-study effects may contribute to the asymmetry observed in the funnel plots (Figure 3). However, the beneficial effect of MSCs on aGVHD mortality was similar between fixed- and random-effects models (Supplementary Table 6), implying that small-study effects did not substantially affect final estimates [64]. Moreover, no study was added in the trim and fill analysis. Thus, the funnel plot asymmetry may have been associated with other types of bias.

## DISCUSSION

To our knowledge, this is the first meta-analysis to evaluate the prophylactic effects of MSCs on aGVHD in animal models of allo-HSCT. This meta-analysis indicates that MSCs significantly prevent mortality and alleviate the clinical manifestations of aGVHD in animals that undergo allo-HSCT. In addition, MSCs provided robust favorable prophylactic effects against aGVHD across recipient species, MSC doses and administration times.

It should be noted that this meta-analysis included only mice and rats because few large animal studies are available. Several clinically relevant parameters, including the MSC source, dose and administration time, may have contributed to the heterogeneity and inconsistent results observed in these studies. We therefore conducted a subgroup meta-analysis based on these factors. One study reported that MSCs suppress immune responses only in an inflammatory environment [65], raising the question of whether co-transplanting MSCs with grafts may prevent aGVHD. The results of the subgroup meta-analysis indicated that co-transplanting MSCs had a prophylactic effect that was similar to the effect of infused MSCs administered more than one day after allo-HSCT. Our subgroup meta-analysis demonstrated that MSCs produced a better prophylactic effect when administered at relatively high doses, consistent with another study that evaluated the effects of increasing doses of MSCs [40]. However, this dose-response effect may not be beneficial when the MSC dose is above a threshold [66]. Furthermore, the results of the subgroup meta-analysis seemed to suggest that differences might be based on the MSC source because favorable prophylactic effects were observed for BM- and UCB-derived MSCs but not adipose tissue- and UC-derived MSCs. Subsequent meta-regression analyses have consistently indicated that the MSC source is a significant contributor to heterogeneity. However, these results should be interpreted with caution because only a small number of relevant studies were included in the analysis.

A number of questions should be considered when translating these results into clinical trials. First, this meta-analysis focused on un-manipulated MSCs. Therefore, whether genetically modified or cytokine pre-treated MSCs are superior to un-manipulated MSCs as aGVHD prophylactics warrants further investigation. Second, cryopreserved MSCs may exert smaller immunomodulatory effects than freshly harvested cells [67]. However, we cannot directly compare cryopreserved MSCs to fresh MSCs because of the limited number of available studies. Third, whether cell senescence impairs the immunomodulatory effects of MSCs remains unclear. Fourth, this meta-analysis exhibited publication bias that was often associated with overestimations of the efficacy of the intervention. We acknowledge this is a limitation of this meta-analysis. Finally, while murine models cannot fully replicate the pathophysiology of human aGVHD [68, 69], such models are useful because they increase our understanding of GVHD and provide a basis for forming clinically translatable ideas [69].

In summary, in this meta-analysis, we show that MSCs significantly prevent mortality and alleviate the clinical manifestations of aGVHD in animal models, supporting further investigations into the use of MSCs as prophylactics against aGVHD in clinical trials.

## MATERIALS AND METHODS

### Search strategy

A systematic literature search of PubMed and the Excerpta Medica Database (Embase) was conducted to identify studies published before February 2016 using the following key word search terms: “mesenchymal stem cell”, “mesenchymal stromal cell”, “MSC”, “graft versus host”, “graft vs host” and “GVHD”. The language was restricted to English. The detailed search criteria are listed in Supplementary Tables 7 and 8. Relevant controlled studies evaluating the prophylactic effect of MSCs on aGVHD in rat or mouse models of allo-HSCT were identified. In addition, the reference lists of all identified studies were manually searched.

### Selection criteria

Two independent researchers evaluated all potentially relevant studies. After titles/abstracts were screened, all suspect articles were submitted to full-text screening to avoid discarding relevant reports. All controlled studies that evaluated the prophylactic efficacy of MSC adoptive transplantation in aGVHD in rat or mouse models of allo-HSCT and that reported aGVHD mortality or aGVHD clinical score outcomes, regardless of animal age, sex or strain, were included. Control interventions included saline, culture medium and no treatment.

Studies using manipulated MSCs (i.e., MSCs genetically modified to overexpress particular molecules or MSCs pre-treated with cytokines) were excluded. Furthermore, studies using MSCs concomitantly with other cell types or other therapies were also excluded. Because we were interested only in the prophylactic effects of MSCs on aGVHD, studies evaluating therapeutic effects of MSCs on established aGVHD were excluded. All discrepancies were resolved by consulting with a specialist.

### Data extraction

Two researchers independently extracted the data. All related data, including reference details (the first author and publication year), donor animals (species and strain), recipient animals (strain, age and sex), graft, sample size, MSC source (donor species and tissue origin), MSC dose, administration time and the above-mentioned outcomes (aGVHD mortality and aGVHD clinical scores), were extracted.

The corresponding authors were contacted if the data were incomplete (i.e., if values for the mean and standard deviations (SD) were not reported). If no response was received from the corresponding authors, the values for the means and SDs were calculated from graphs in the original articles using digital ruler software. If a single study compared different MSC doses and/or administration times using one control group, the data were treated as independent comparison experiments, and the control group was divided by the number of experimental groups to ensure that the total number of controls was not changed [70]. If aGVHD clinical scores were serially monitored, only the data for the time point at which the most severe aGVHD manifestations developed were extracted.

### Methodological quality evaluation

Two researchers independently assessed the methodological quality of each included study using previously reported criteria [70], with slight modifications. These criteria included seven items: peer-reviewed publication, randomization, concealment of allocation, blindness to outcome assessors, estimation of sample size, compliance with animal welfare requirements and conflict of interest statements.

### Statistical analysis

Statistical analyses were performed using Stata software (version 12.0, Stata Corporation, College Station, TX, USA). A risk ratio (RR) and 95% confidence interval (CI) were used to pool the aGVHD mortality data. Furthermore, because the clinical scoring system differed between studies, the standardized mean difference (SMD) and 95% CI were used to pool the aGVHD clinical score data. Statistical heterogeneity among studies was assessed using the I^2^ statistic. Values of I^2^ > 50% and *P* < 0.1 indicated significant heterogeneity among the studies. Next, to reveal the potential source of statistical heterogeneity, we conducted a subgroup meta-analysis and univariate meta-regression based on the following clinical variables: recipient species, MSC source, MSC dose and administration time. The DerSimonian and Laird random-effects model was used to provide more conservative conclusions when anticipated significant heterogeneity was identified among the included studies [71]. Funnel plots were constructed to examine the potential publication bias [72]. If funnel plot asymmetry was found, Egger's tests were conducted to confirm the existence of publication bias [73], and the asymmetry was adjusted using the Duval and Tweedie trim and fill analysis [74]. A *P* value of less than 0.05 was considered statistically significant.

## SUPPLEMENTARY FIGURE AND TABLES








