# Supplementary material for: Mesenchymal stem cells provide prophylaxis against acute graft-versus-host disease following allogeneic hematopoietic stem cell transplantation: A meta-analysis of animal models
Source: Oncotarget. 2016 Aug 12;7(38):61764–74. doi: 10.18632/oncotarget.11238 (PMC5308689; doi:10.18632/oncotarget.11238)
Supplement: Supplementary file 1 [file oncotarget-07-61764-s001.pdf]

Mesenchymal stem cells provide prophylaxis against acute graft-versus-host disease following allogeneic hematopoietic stem cell transplantation: A meta-analysis of animal models

SUPPLEMENTARY FIGURE AND TABLES

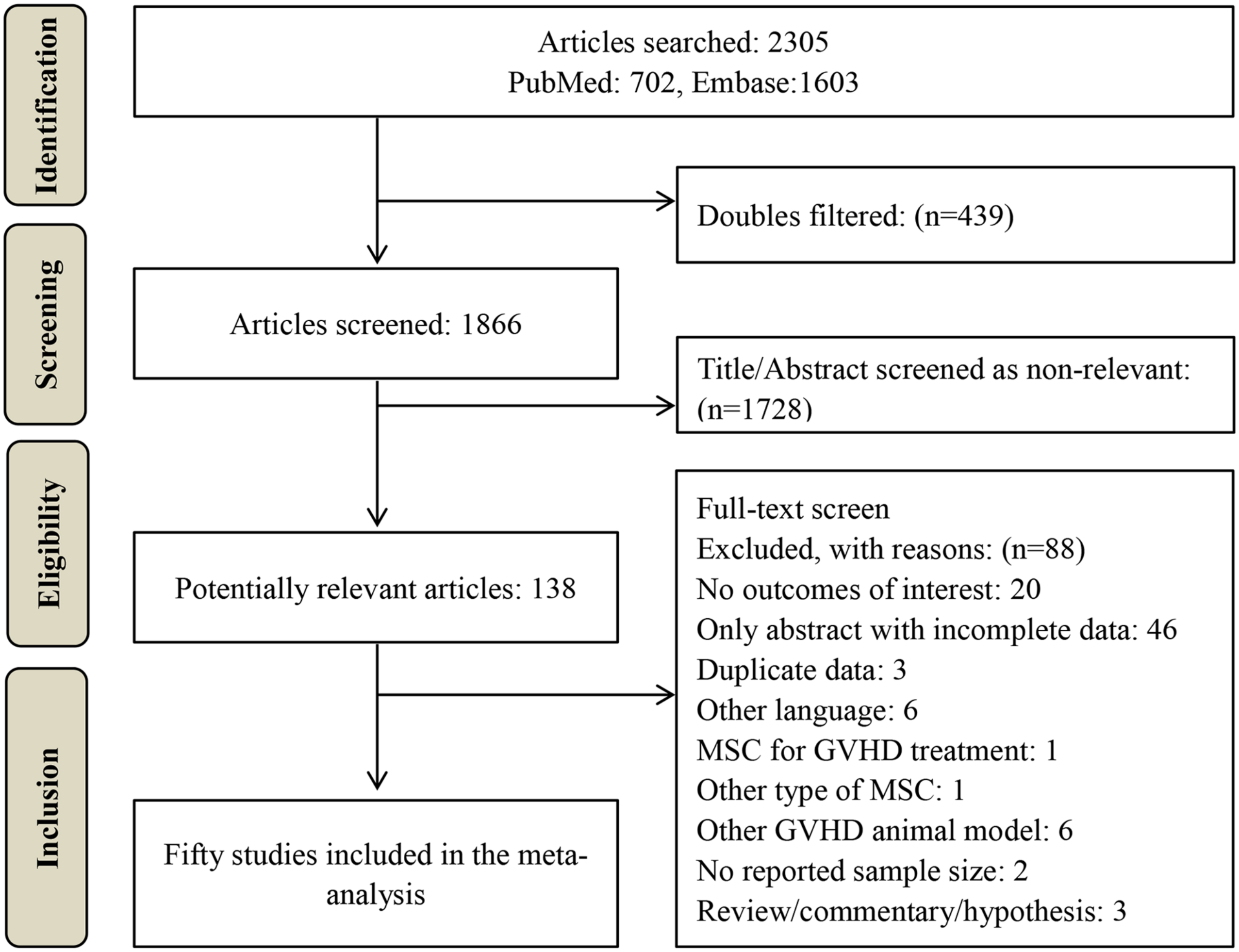

Supplementary Figure 1: Flowchart of the systematic search used in this study. MSC: mesenchymal stem cell, GVHD: graft-versus-host disease.

Supplementary Table 1: Characteristics of excluded full-text studies

See Supplementary File 1

Supplementary Table 2: Characteristics of the included studies

See Supplementary File 2

Supplementary Table 3: Methodological quality of the included studies

See Supplementary File 3

Supplementary Table 4: Subgroup meta-analysis and meta-regression (aGVHD mortality)

Subgroup meta-analysis	Number of comparisons	Risk ratio (RR) (95% confidence interval, CI)	I^2, P value	Univariate meta-regression Adjusted R^2
**Recipient species**				
Rat	6	0.58 (0.39, 0.86)	42.0%, 0.125	0.36%
Mouse	81	0.71 (0.63, 0.80)	66.7%, < 0.001	
**MSC source**				
Rat BM	6	0.58 (0.39, 0.86)	42.0%, 0.125	5.41%
Mouse BM	44	0.77 (0.65, 0.91)	68.1%, < 0.001	
Human BM	13	0.68 (0.51, 0.93)	76.3%, < 0.001	
Human UCB	8	0.56 (0.37, 0.85)	52.4%, 0.040	
Human UC	4	0.51 (0.20, 1.31)	64.0%, 0.040	
Mouse adipose tissue	3	0.49 (0.23, 1.06)	79.4%, 0.008	
**MSC dose**				
0.02 to 0.5 million	43	0.75 (0.65, 0.86)	58.3%, < 0.001	1.73%
1 to 5 million	36	0.66 (0.52, 0.83)	73.9%, < 0.001	
10 to 20 million	2	0.41 (0.23, 0.75)	0.0%, 0.505	
**MSC administration time**				
Co-transplantation with allo-HSCT	40	0.69 (0.57, 0.83)	73.8%, < 0.001	0.27%
Multiple doses including co-transplantation	18	0.75 (0.57, 0.98)	69.0%, < 0.001	
Single or multiple doses, 1 day post-allo-HSCT	23	0.69 (0.56, 0.84)	45.6%, 0.010	

Note: the DerSimonian and Laird random-effects model was used to pool data. Abbreviations: MSCs: mesenchymal stem cells, BM: bone marrow, UCB: umbilical cord blood, UC: umbilical cord.

Supplementary Table 5: Subgroup meta-analysis and meta-regression (aGVHD clinical scores)

Subgroup meta-analysis	Number of comparisons	Standardized mean difference (SMD) (95% CI)	I^2, P value	Univariate meta-regression Adjusted R^2
**Recipient species**				
Rat	2	-2.44 (-4.47, -0.40)	78.7%, 0.030	-3.87%
Mouse	39	-3.70 (-4.58, -2.82)	93.1%, < 0.001	
**MSC source**				
Rat BM	2	-2.44 (-4.47, -0.40)	78.7%, 0.030	-2.76%
Mouse BM	25	-3.07 (-4.25, -1.88)	94.3%, < 0.001	
Human BM	3	-5.44 (-9.02, -1.86)	88.0%, < 0.001	
Human UCB	6	-4.88 (-6.53, -3.23)	72.3%, 0.003	
Human adipose tissue	2	-3.85 (-8.88, 1.18)	89.6%, 0.002	
**MSC dose**				
0.02 to 0.5 million	18	-3.24 (-4.58, -1.90)	93.2%, < 0.001	-4.58%
1 to 5 million	19	-4.16 (-5.46, -2.85)	93.7%, < 0.001	
10 to 20 million	2	-4.37 (-7.07, -1.67)	76.7%, 0.038	
**MSC administration time**				
Co-transplantation with allo-HSCT	23	-3.56 (-4.66, -2.47)	93.5%, < 0.001	-4.05%
Multiple doses including co-transplantation	8	-3.15 (-5.70, -0.60)	94.5%, < 0.001	
Single or multiple doses, 1 day post-allo-HSCT	8	-5.14 (-7.31, -2.96)	91.2%, < 0.001	

Note: the DerSimonian and Laird random-effects model was used to pool data. Abbreviations: MSCs: mesenchymal stem cells, BM: bone marrow, UCB: umbilical cord blood.

Supplementary Table 6: Sensitivity analysis

	Intervention effect estimate	P value
**aGVHD mortality**		
Fixed-effects model	RR: 0.64, 95% CI: 0.59 ~ 0.68	2.19×10^{-36}
Random-effects model	RR: 0.70, 95% CI: 0.62 ~ 0.79	2.73×10^{-9}
**aGVHD clinical scores**		
Fixed-effects model	SMD: -1.87, 95% CI: -2.07 ~ -1.67	3.14×10^{-75}
Random-effects model	SMD: -3.60, 95% CI: -4.43 ~ -2.76	3.61×10^{-17}

Abbreviations: aGVHD: acute graft-versus-host disease, RR: risk ratio, CI: confidence interval, SMD: standardized mean difference.

Supplementary Table 7: Search criterion for PubMed (from inception to Feb 29, 2016)

No.	Query Results	Results
#20	#14 AND #19	702
#19	#15 OR #16 OR #17 OR #18	29642
#18	Search "Graft vs Host Disease"[Mesh]	18898
#17	Search GVHD[Title/Abstract]	13161
#16	Search graft versus host[Title/Abstract]	19863
#15	Search graft vs host[Title/Abstract]	2430
#14	#1 OR #2 OR #3 OR #4 OR #5 OR #6 OR #7 OR #8 OR #9 OR #10 OR #11 OR #12 OR #13	44010
#13	Search "Mesenchymal Stromal Cells" [Mesh]	20783
#12	Search MSC*[Title/Abstract]	22372
#11	Search adipose stem cells[Title/Abstract]	376
#10	Search adipose stem cell[Title/Abstract]	101
#9	Search marrow stromal cells[Title/Abstract]	5218
#8	Search marrow stromal cell[Title/Abstract]	1261
#7	Search wharton's jelly[Title/Abstract]	649
#6	Search mesenchymal precursor cells[Title/Abstract]	183
#5	Search mesenchymal precursor cell[Title/Abstract]	34
#4	Search mesenchymal stromal cells[Title/Abstract]	3336
#3	Search mesenchymal stromal cell[Title/Abstract]	621
#2	Search mesenchymal stem cells[Title/Abstract]	23456
#1	Search mesenchymal stem cell[Title/Abstract]	5401

Supplementary Table 8: Search criterion of Embase (from inception to Feb 29, 2016)

No.	Query Results	Results
#21	#14 AND #20	1603
#20	#15 OR #16 OR #17 OR #18 OR #19	58029
#19	‘acute graft versus host disease’/exp	6431
#18	‘graft versus host reaction’/exp	49571
#17	gvhd:ab,ti	23915
#16	graft:ab,ti AND versus:ab,ti AND host:ab,ti	28380
#15	graft:ab,ti AND vs:ab,ti AND host:ab,ti	5762
#14	#1 OR #2 OR #3 OR #4 OR #5 OR #6 OR #7 OR #8 OR #9 OR #10 OR #11 OR #12 OR #13	78108
#13	‘mesenchymal stroma cell’/exp	6112
#12	msc*:ab,ti	33557
#11	adipose:ab,ti AND stem:ab,ti AND cells:ab,ti	8524
#10	adipose:ab,ti AND stem:ab,ti AND cell:ab,ti	6495
#9	marrow:ab,ti AND stromal:ab,ti AND cells:ab,ti	17728
#8	marrow:ab,ti AND stromal:ab,ti AND cell:ab,ti	14304
#7	wharton:ab,ti AND jelly:ab,ti	995
#6	mesenchymal:ab,ti AND precursor:ab,ti AND cells:ab,ti	1758
#5	mesenchymal:ab,ti AND precursor:ab,ti AND cell:ab,ti	1411
#4	mesenchymal:ab,ti AND stromal:ab,ti AND cells:ab,ti	11170
#3	mesenchymal:ab,ti AND stromal:ab,ti AND cell:ab,ti	9062
#2	mesenchymal:ab,ti AND stem:ab,ti AND cells:ab,ti	42674
#1	mesenchymal:ab,ti AND stem:ab,ti AND cell:ab,ti	33696
