# Supplementary material for: Mesenchymal stem cells provide prophylaxis against acute graft-versus-host disease following allogeneic hematopoietic stem cell transplantation: A meta-analysis of animal models
Source: Oncotarget. 2016 Aug 12;7(38):61764–74. doi: 10.18632/oncotarget.11238 (PMC5308689; doi:10.18632/oncotarget.11238)
Supplement: Supplementary file 2 [file oncotarget-07-61764-s002.docx]

**Table S1. Characteristics of excluded full-text studies**

| **Studies** | **Reasons for exclusion** |
| --- | --- |
| Miyashima 1996 ^[1]^ | Only abstract with incomplete data |
| Maitra 2004 ^[2]^ | No outcomes of interest |
| Hu 2005 ^[3]^ | Other language |
| Tian 2006 ^[4]^ | Duplicate data |
| Nauta 2006 ^[5]^ | No outcomes of interest |
| Krampera 2006 ^[6]^ | No outcomes of interest |
| Hu 2007 ^[7]^ | Other language |
| Li 2007 ^[8]^ | No reported sample sizes |
| Huang 2007 ^[9]^ | Other language |
| Miyake 2008 ^[10]^ | No outcomes of interest |
| Kao 2009 ^[11]^ | Only abstract with incomplete data |
| Shi 2009 ^[12]^ | Only abstract with incomplete data |
| Bruck 2009 ^[13]^ | Only abstract with incomplete data |
| Szilagyi 2010 ^[14]^ | Allogeneic heart transplantation |
| Rae 2010 ^[15]^ | Skin explant model |
| Oviedo 2010 ^[16]^ | Only abstract with incomplete data |
| Li 2010 ^[17]^ | No outcomes of interest |
| Kuci 2010 ^[18]^ | No outcomes of interest (only reported hematopoietic engraftment-promoting ability) |
| Fan 2010 ^[19]^ | Only abstract with incomplete data |
| Jeon 2010 ^[20]^ | No reported sample sizes |
| Ezzelarab 2010 ^[21]^ | Commentary |
| Bao 2010 ^[22]^ | Other language |
| Li 2011^[23]^ | Other language |
| Mielcarek 2011 ^[24]^ | Dog graft-versus-host disease model |
| Auletta 2011 ^[25]^ | Only abstract with incomplete data |
| Luyckx 2011 ^[26]^ | No outcomes of interest |
| Solchaga 2011 ^[27]^ | No outcomes of interest (only *ex vivo* experiments) |
| Zinocker 2011 ^[28]^ | Mesenchymal stromal cells harboring *Mycoplasma hyorhinis* |
| Zinöcker 2011 ^[29]^ | Only abstract with incomplete data |
| Lopez-Rodriguez 2011 ^[30]^ | Only abstract with incomplete data |
| Zhang 2011 ^[31]^ | No outcomes of interest |
| Cho 2011 ^[32]^ | Only abstract with incomplete data |
| Capelli 2011 ^[33]^ | Only abstract with incomplete data |
| Yao 2011 ^[34]^ | Only abstract with incomplete data |
| Yang 2011 ^[35]^ | No outcomes of interest |
| Taghizadeh 2011 ^[36]^ | No outcomes of interest |
| Gregoire-Gauthier 2011 ^[37]^ | Only abstract with incomplete data |
| Lee 2011 ^[38]^ | Only abstract with incomplete data |
| Hinden 2011 ^[39]^ | Only abstract with incomplete data |
| Hilger 2011 ^[40]^ | Only abstract with incomplete data |
| Joo 2011 ^[41]^ | No outcomes of interest |
| Jang 2011 ^[42]^ | Only abstract with incomplete data |
| Hu 2011 ^[43]^ | No outcomes of interest |
| Capelli 2011 ^[44]^ | Chronic graft-versus-host disease model |
| Asari 2011 ^[45]^ | No outcomes of interest |
| Colita 2012 ^[46]^ | Medical hypotheses |
| Zheng 2012 ^[47]^ | Only abstract with incomplete data |
| Yang 2012 ^[48]^ | Only abstract with incomplete data |
| Wang 2012 ^[49]^ | Only abstract with incomplete data |
| Kornblit 2013 ^[50]^ | Dog bone marrow transplantation model |
| Hinden 2013 ^[51]^ | Only abstract with incomplete data |
| Köberle 2013 ^[52]^ | Only abstract with incomplete data |
| Ma 2013 ^[53]^ | Only abstract with incomplete data |
| O'Kane 2013 ^[54]^ | Only abstract with incomplete data |
| Qiao 2013 ^[55]^ | Only abstract with incomplete data |
| Wu 2013 ^[56]^ | No outcomes of interest (only reported hematopoiesis-enhancing ability) |
| Wuttisarnwattana 2013^[57]^ | No outcomes of interest |
| Fan 2013 ^[58]^ | No outcomes of interest |
| Zheng 2013 ^[59]^ | Only abstract with incomplete data |
| Ringdén 2013 ^[60]^ | Only abstract with incomplete data |
| Lim 2013 ^[61]^ | Only abstract with incomplete data |
| Liu 2014 ^[62]^ | Review |
| Li 2014 ^[63]^ | Duplicate data |
| Fujino 2014 ^[64]^ | Duplicate data |
| Chen 2014 ^[65]^ | Other language |
| Normanton 2014 ^[66]^ | No outcomes of interest |
| Holubova 2014 ^[67]^ | No outcomes of interest (only *in vitro* experiments) |
| Zheng 2014 ^[68]^ | Only abstract with incomplete data |
| Chen 2014 ^[69]^ | Only abstract with incomplete data |
| Chen 2014 ^[70]^ | Only abstract with incomplete data |
| Yamahara 2014 ^[71]^ | No outcomes of interest (only reported weight loss) |
| Cho 2014 ^[72]^ | Only abstract with incomplete data |
| Im 2014 ^[73]^ | Only abstract with incomplete data |
| Sadeghi 2014 ^[74]^ | Only abstract with incomplete data |
| Roux 2014 ^[75]^ | Only abstract with incomplete data |
| Robles 2014 ^[76]^ | Only abstract with incomplete data |
| Auletta 2014 ^[77]^ | Only abstract with incomplete data |
| Heider 2014 ^[78]^ | Only abstract with incomplete data |
| Barrett 2014 ^[79]^ | Only abstract with incomplete data |
| Wen 2015 ^[80]^ | Only abstract with incomplete data |
| Wang 2015 ^[81]^ | Only abstract with incomplete data |
| Sanchez-Abarca 2015 ^[82]^ | Ocular graft-versus-host disease model |
| Roux 2015 ^[83]^ | Only abstract with incomplete data |
| Ringdén 2015 ^[84]^ | Only abstract with incomplete data |
| Nagamura-Inoue 2015 ^[85]^ | Only abstract with incomplete data |
| Jiang 2015 ^[86]^ | Only abstract with incomplete data |
| Gu 2015 ^[87]^ | Only abstract with incomplete data |
| Amarnath 2015 ^[88]^ | MSC administration to treat established GVHD |

**REFERENCES**

1. Miyashima S, Nagata N, Nakagawa T, Hosaka N, Takeuchi K, Ogawa R, Ikehara S. Prevention of lpr-graft-versus-host disease and transfer of autoimmune diseases in normal C57BL/6 mice by transplantation of bone marrow cells plus bones (stromal cells) from MRL/lpr mice. J Immunol. 1996; 156: 79-84.

2. Maitra B, Szekely E, Gjini K, Laughlin MJ, Dennis J, Haynesworth SE, Koç ON. Human mesenchymal stem cells support unrelated donor hematopoietic stem cells and suppress T-cell activation. Bone Marrow Transplant. 2004; 33: 597-604.

3. Hu WB, Gao QP, Chen YH. [Effect of bone marrow mesenchymal stem cells on acute graft versus host disease and graft versus leukemia after allogeneic bone marrow transplantation]. Zhongguo Shi Yan Xue Ye Xue Za Zhi. 2005; 13: 404-407.

4. Tian Y, Deng YB, Huang YJ, Na XD, Li Y, Ye MH. [Role of bone marrow-derived mesenchymal stem cells in reduction of graft-versus-host disease by effecting CD4+CD25+ regulatory T cells in rats]. Zhongguo Shi Yan Xue Ye Xue Za Zhi. 2006; 14: 1210-1214.

5. Nauta AJ, Westerhuis G, Kruisselbrink AB, Lurvink EG, Willemze R, Fibbe WE. Donor-derived mesenchymal stem cells are immunogenic in an allogeneic host and stimulate donor graft rejection in a nonmyeloablative setting. Blood. 2006; 108: 2114-2120.

6. Krampera M, Cosmi L, Angeli R, Pasini A, Liotta F, Andreini A, Santarlasci V, Mazzinghi B, Pizzolo G, Vinante F, et al. Role for interferon-gamma in the immunomodulatory activity of human bone marrow mesenchymal stem cells. Stem Cells. 2006; 24: 386-398.

7. Hu KX, Zhao SF, Sun QY, Guo M, Ai HS. [Effect of bone marrow mesenchymal stem cells on immunoregulation in H-2 haploidentical bone marrow transplantation mice]. Zhonghua Xue Ye Xue Za Zhi. 2007; 28: 505-509.

8. Li H, Guo ZK, Li XS, Hou CM, Tang PH, Mao N. Functional and phenotypic alteration of intrasplenic lymphocytes affected by mesenchymal stem cells in a murine allosplenocyte transfusion model. Cell Transplant. 2007; 16: 85-95.

9. Huang K, Huang SL, Zhou DH, Cai Y, Zhang XC, Li Y. [Experiment study of efficacy on hematopoietic reconstitution and GVHD prophylaxis after mesenchymal cell infused by intra-bone marrow cavity or intravenous in rat BMT models]. Zhonghua Xue Ye Xue Za Zhi. 2007; 28: 87-92.

10. Miyake T, Inaba M, Fukui J, Ueda Y, Hosaka N, Kamiyama Y, Ikehara S. Prevention of graft-versus-host disease by intrabone marrow injection of donor T cells: involvement of bone marrow stromal cells. Clin Exp Immunol. 2008; 152: 153-162.

11. Kao YH, Tsai BY, Chiang BL. The immunosuppressive effects of human mesenchymal stem cells in human graft-versus-host disease-like syndrome of NOD/SCID mice. Eur J Immunol. 2009; 39: S516.

12. Shi Y, Zhang L, Zhao X, Xu G, Zhang Y, Roberts AI, Zhao RC, Ren G. Inflammatory cytokine induced immunosuppression in mesenchymal stem cells. FASEB J. 2009; 23: 297-304.

13. Bruck F, Lechanteur C, De Leval L, Humblet-Baron S, Stephan R, Gothot A, Beguin Y, Baron F. IP Co-transplantation of mesenchymal stem cells (MSC) failed to prevent acute GVHD in a humanized MICE model. Experimental Hematology. 2009; 37: S87-S88.

14. Szilagyi E, Douglas GW, Polchert D, Genrich K, Bartholomew A. Reprogramming mesenchymal stem cells for increased tolerogenic capacity. Am J Transplant. 2010; 10: 561.

15. Rae M, Wang XN, Collin M, Dickinson A. A preliminary investigation into the kinetics of MSC induced immuno-modulation during the allo-reactive phase of an MLR. Bone Marrow Transplant. 2010; 45: S301-S302.

16. Oviedo A, Yañez R, Aldea M, Rubio A, Bueren J, Lamana M. Effect of mesenchymal stromal cells on GVHD and GVL reactions in a CML relapse mouse model. Hum Gene Ther. 2010; 21: 1449.

17. Li H, Guo Z, Zhu H, Li XS, Jiang X, Yao H, Wang X, Liao L, Liu Y, Wu Y, Zhang Y, Mao N. Transplanted mesenchymal stem cells can inhibit the three developmental stages of murine acute graft-versus-host disease. In Vivo. 2010; 24: 659-666.

18. Kuçi S, Kuçi Z, Kreyenberg H, Deak E, Pütsch K, Huenecke S, Amara C, Koller S, Rettinger E, Grez M, et al. CD271 antigen defines a subset of multipotent stromal cells with immunosuppressive and lymphohematopoietic engraftment-promoting properties. Haematologica. 2010; 95: 651-659.

19. Fan H. Xenoreactivity of human umbilical cord derived-mesenchymal stem cells in a MHC-mismatched allogeneic graft-versus-host disease murine model. Vox Sang. 2010; 99: 61-62.

20. Jeon MS, Lim HJ, Yi TG, Im MW, Yoo HS, Choi JH, Choi EY, Song SU. Xenoreactivity of human clonal mesenchymal stem cells in a major histocompatibility complex-matched allogeneic graft-versus-host disease mouse model. Cell Immunol. 2010; 261: 57-63.

21. Ezzelarab M, Ayares D, Cooper DKC. The potential of genetically-modified pig mesenchymal stromal cells in xenotransplantation. Xenotransplantation. 2010; 17: 3-5.

22. Bao XC, Wang JM, Zhang WP, Zhou H, Zheng XL, Gao L. [The role of marrow derived mesenchymal stem cells in the prevention and treatment of acute graft-versus-host in mice.]. Zhonghua Xue Ye Xue Za Zhi. 2010; 31: 108-113.

23. Li ZY, Wang CQ, Lu G. [Effects of bone marrow mesenchymal stem cells on hematopoietic recovery and acute graft-versus-host disease in murine allogeneic umbilical cord blood transplantation model]. Zhonghua Xue Ye Xue Za Zhi. 2011; 32: 786-789.

24. Mielcarek M, Storb R, Georges GE, Golubev L, Nikitine A, Hwang B, Nash RA, Torok-Storb B. Mesenchymal stromal cells fail to prevent acute graft-versus-host disease and graft rejection after dog leukocyte antigen-haploidentical bone marrow transplantation. Biol Blood Marrow Transplant. 2011; 17: 214-225.

25. Auletta JJ, Eid S, Keller M, Metheny L, Guardia-Wolff R, Lee Z, Solchaga LA, Cooke KR. Human mesenchymal stem cells attenuate graft-versus-host disease and maintain graft-versus-leukemia in murine allogeneic bone marrow transplantation. Blood. 2011; 118: 1907.

26. Luyckx A, De Somer L, Jacobs S, Rutgeerts O, Lenaerts C, Roobrouck VD, Verfaillie CM, Waer M, Van Gool SW, Billiau AD. Oct4-negative multipotent adult progenitor cells and mesenchymal stem cells as regulators of T-cell alloreactivity in mice. Immunol Lett. 2011; 137: 78-81.

27. Auletta JJ, Zale EA, Welter JF, Solchaga LA. Fibroblast growth factor-2 enhances expansion of human bone marrow-derived mesenchymal stromal cells without diminishing their immunosuppressive potential. Stem Cells Int. 2011; 2011: 235176.

28. Zinöcker S, Wang MY, Gaustad P, Kvalheim G, Rolstad B, Vaage JT. Mycoplasma contamination revisited: mesenchymal stromal cells harboring mycoplasma hyorhinis potently inhibit lymphocyte proliferation in vitro. PLOS ONE. 2011; 6: e16005.

29. Zinöcker S, Vaage JT. Rat bone marrow stromal cells suppress T cell activation by production of nitric oxide. Scand J Immunol. 2011; 73: 379.

30. Lopez-Rodriguez Y, Trevino E, Weiss ML. Wharton's jelly mesenchymal stromal cells (WJCS) as immunoregulators in allogeneic transplantation. Placenta. 2011; 32: S329.

31. Zhang C, Chen XH, Zhang X, Gao L, Gao L, Kong PY, Peng XG, Sun AH, Wang QY. 2011. Regulation of acute graft-versus-host disease by human umbilical cord blood derived stromal cells in haploidentical stem cell transplantation in mice through very late activation antigen-4. Clin Immunol. 2011; 139: 94-101.

32. Cho SG, Lim JY, Jeon E, et al. Combination cell therapy of ex-vivo expanded regulatory t cells and human adipose tissue-derived MSCs effectively inhibits acute graft-versus-host disease in murine model. Haematologica. 2011; 96: 193.

33. Capelli C, Gotti E, Morigi M, Rota C, Weng L, Dazzi F, Spinelli O, Rambaldi A, Golay J, Introna M. Massive expansion of umbilical cord derived mesenchymal stromal cells (MSC): an innovative and unlimited source of MSC with potent immunosuppressive activity for the treatment of GvHD. Bone Marrow Transplant. 2011; 46: S314.

34. Yao M, Chou SH, Lee W, Chen YC, Tang JL, Chen YC, Ko BS. Placental-derived stem cells improve haematopoiesis and immune-tolerance after allogeneic haematopoietic stem cell transplantation in murine models. Bone Marrow Transplant. 2011; 46: S301-S302.

35. Yang HM, Cho MR, Sung JH, Yang SJ, Nam MH, Roh CR, Kim JM, Shin M, Song SH, Kwon CH, Joh JW, Kim SJ. The effect of human fetal liver-derived mesenchymal stem cells on CD34+ hematopoietic stem cell repopulation in NOD/Shi-scid/IL-2Ra(null) mice. Transplant Proc. 2011; 43: 2004-2008.

36. Taghizadeh RR, Pollok KE, Betancur M, Boissel L, Cetrulo KJ, Marino T, Wolfberg A, Klingemann HG, Cetrulo CL. Wharton's jelly derived mesenchymal stem cells: regenerative medicine beyond umbilical cord blood. Placenta. 2011; 32: S339.

37. Gregoire-Gauthier J, Selleri S, Fontaine F, Haddad E. Mesenchymal stromal cells can prevent graft-versus-host disease in a NOD/SCID γc-xenogeneic mouse model. Pediatr Transplant. 2011; 15: 128–141.

38. Lee MW, Kim DS, Kim HJ, Son MH, Lee SH, Jung HL, Yoo KH, Sung KW, Koo HH. Induction of indoleamine 2,3-dioxygenase expression by interferon-g in human mesenchymal stem cells inhibits graft versus host disease. Blood. 2011; 118.

39. Hinden L, Almogi-Hazan O, Aker M, Or R. Immuneregulation of graft-versus-host disease in bone marrow transplanted mice using activated mesenchymal cells and kynurenine. Bone Marrow Transplant. 2011; 46: S242.

40. Hilger N, Ackermann M, Tuche S, Jahns J, Emmrich F, Sack U, Fricke S. New stem cell source: nonadherent bone marrow cells facilitate hematopeisis after hematopoeitic stem cell transplantation in humanized mice. Cytometry B Clin Cytometry. 2011; 80B: 405-406.

41. Joo SY, Cho KA, Jung YJ, Kim HS, Park SY, Choi YB, Hong KM, Woo SY, Seoh JY, Ryu KH. Bioimaging for the monitoring of the in vivo distribution of infused mesenchymal stem cells in a mouse model of the graft-versus-host reaction. Cell Biol Int. 2011; 35: 417-421.

42. Jang MJ, Oh SY, Kim H, Shin H, Kim GJ, Chong SY, Oh D, Chung H. Immunosupressive properties of human placenta-derived mesenchymal stem cells on control of acute graft-versus-host disease in mice. J Clin Oncol. 2011; 29: 6547.

43. Hu KX, Wang MH, Fan C, Wang L, Guo M, Ai HS. CM-DiI labeled mesenchymal stem cells homed to thymus inducing immune recovery of mice after haploidentical bone marrow transplantation. Int Immunopharmacol. 2011; 11: 1265-1270.

44. Capelli C, Gotti E, Morigi M, Rota C, Weng L, Dazzi F, Spinelli O, Cazzaniga G, Trezzi R, Gianatti A, Rambaldi A, Golay J, Introna M. Minimally manipulated whole human umbilical cord is a rich source of clinical-grade human mesenchymal stromal cells expanded in human platelet lysate. Cytotherapy. 2011; 13: 786-801.

45. Asari S, Itakura S, Rawson J, Ito T, Todorov I, Nair I, Shintaku J, Liu CP, Kandeel F, Mullen YS. Mesenchymal stem cells facilitate mixed hematopoietic chimerism induction and prevent onset of diabetes in nonobese diabetic mice. Pancreas. 2011; 40: 846-854.

46. Coliţă A, Coliţă A, Zamfirescu D, Lupu AR. Combined procedure of vascularized bone marrow transplantation and mesenchymal stem cells graft - an effective solution for rapid hematopoietic reconstitution and prevention of graft-versus-host disease. Med Hypotheses. 2012; 79: 302-303.

47. Zheng SG, Chen M, Su W, Wang J, Brand D, Ryffel B, He X, Le A. Gingiva-derived mesenchymal stem cells-mediated therapeutic intervention for experimental arthritis through Foxp3+ Tregs. J Immunol. 2012; 188 (1 Suppl.): 56-57.

48. Yang D, Wang JM, Wang LP, Zhou H. Inducible costimulator gene transduced bone marrow derived mesenchymal stem cells attenuate the severity of acute graft-versus-host disease in a mouse haploidentical model. Blood. 2012; 120: 2996.

49. Wang J, Ma D, Li Y, Fang Q, Chen S, Sun J. Lentiviral-mediated ho-1 gene transduced bone mesenchymal stem cells protects against acute graft-versus-host disease in vitro and vivo. Blood. 2012; 120: 4675.

50. Kornblit B, Leisenring WM, Santos EB, Storb R, Sandmaier BM. Safety of treatment with DLA-identical or unrelated mesenchymal stromal cells in DLA-identical canine bone marrow transplantation. Chimerism. 2013; 4: 95-101.

51. Hinden L, Almogi-Hazan O, Or R. Kynurenine regulates the function of bone marrowderived MSCs through aryl hydrocarbon receptor signaling. Hum Gene Ther. 2013; 24: A10.

52. Köberle M, Rodewohl A, Scholbach J, et al. A humanized mouse model of graft-versus-host disease in nod-SCID IL2Rg(null) mice. Regen Med. 2013; 8: 326.

53. Wang J, Li Y, Fang Q, Sun J, Hu X. Ho-1 activation in bone-mesenchymal stem cells for treatment and prevention of acute graft-versus-host disease in mice. Blood. 2013; 122: 5427.

54. O'Kane BJ, Devetten M, Jackson J, Lacy J, Sharp JG. Leukemic relapse following MMSC rescue in a murine model of GVHD. FASEB J. 2013; 27 (1 Suppl.): lb52.

55. Qiao S, Shi Y, Liu W, Yuan J. Allogeneic compact bone-derived mesenchymal stem cell transplantation attenuates the severity of idiopathic pneumonia syndrome in a murine bone marrow transplantation model. Blood. 2013; 122: 4466.

56. Wu KH, Tsai C, Wu HP, Sieber M, Peng CT, Chao YH. Human application of ex vivo expanded umbilical cord-derived mesenchymal stem cells: enhance hematopoiesis after cord blood transplantation. Cell Transplant. 2013; 22: 2041-2051.

57. Wuttisarnwattana P, Wilson DL, Cooke KR, et al. Mesenchymal stem cell immunomodulation effects on graft-versus-host disease as determined with cryo-imaging. Mol Imaging Biol. 2013; 15: S389-S390.

58. Fan X, Gay FP, Ong SY, Ang JM, Chu PP, Bari S, Lim TK, Hwang WY. Mesenchymal stromal cell supported umbilical cord blood ex vivo expansion enhances regulatory T cells and reduces graft versus host disease. Cytotherapy. 2013; 15: 610-619.

59. Zheng SG, Chen M, Chen DD, et al. Human gingiva-derived mesenchymal stromal cells inhibit graft-versus-host disease through CD39 and IDO. Arthritis Rheum. 2013; 65: S789.

60. Ringdén O, Kaipe H, Erkers T, Nava S, Molldén P, Sadeghi B. Decidual stromal cells from placenta-experimental and clinical studies. Cell J. 2013; 15: 13.

61. Lim J, Kim E, Im K, Kim N, Kim T, Sohn H, Choi J, Cho S. Combination cell therapy using mesenchymal stem cells and regulatory t cells provides a synergistic immunomodulatory effect associated with reciprocal regulation of TH1/TH2 and TH17/TREG cells in a murine acute graft-versus-host disease model. Cytotherapy. 2013; 15: S39.

62. Liu SS, Zhang C, Zhang X, Chen XH. Human umbilical cord blood-derived stromal cells: A new source of stromal cells in hematopoietic stem cell transplantation. Crit Rev Oncol Hematol. 2014; 90: 93-98.

63. Li H, Jiang YM, Sun YF, Li P, Dang RJ, Ning HM, Li YH, Zhang YJ, Jiang XX, Guo XM, et al. CCR7 expressing mesenchymal stem cells potently inhibit graft-versus-host disease by spoiling the fourth supplemental Billingham's tenet. PLOS ONE. 2014; 9: e115720.

64. Fujino M, Zhu P, Kitazawa Y, Chen JM, Zhuang J, Li XK. Mesenchymal stem cells attenuate rat graft-versus-host disease. Methods Mol Biol. 2014; 1213: 341-353.

65. Chen W, Li M, Zhang C, Wang X, Pan B, Zeng L, Li Z, Xu K. [Effect of lentiviral vector mediated CXCR4 gene overexpressed mesenchymal stem cell on the protection of mice against graft-versus-host disease]. Zhonghua Xue Ye Xue Za Zhi. 2014; 35: 936-940.

66. Normanton M, Alvarenga H, Hamerschlak N, Ribeiro A, Kondo A, Rizzo LV, Marti LC. Interleukin 7 plays a role in T lymphocyte apoptosis inhibition driven by mesenchymal stem cell without favoring proliferation and cytokines secretion. PLOS ONE. 2014; 9: e106673.

67. Holubova M, Lysak D, Vlas T, Vannucci L, Jindra P. Expanded cryopreserved mesenchymal stromal cells as an optimal source for graft-versus-host disease treatment. Biologicals. 2014; 42: 139-144.

68. Zheng SG, Chen M, Wang J, Olsen N. Human gingiva-derived mesenchymal stromal cells inhibit graft-versus-host disease through CD39 and IDO. J Immunol. 2014; 192: 200-223.

69. Chen M, Guo Z, Wang D, Ju W, Gu J, Zadeh HH, Zheng S, He X. Human gingiva-derived mesenchymal stromal cells inhibit graft-versus-host disease through CD39 and IDO. Liver Transpl. 2014; 20: S139.

70. Chen M, Gu J, Wang D, et al. Human gingiva-derived mesenchymal stromal cells inhibit graft-versus-host disease through CD39 and IDO. Transplantation. 2014; 98: 416.

71. Yamahara K, Harada K, Ohshima M, Ishikane S, Ohnishi S, Tsuda H, Otani K, Taguchi A, Soma T, Ogawa H, et al. Comparison of angiogenic, cytoprotective, and immunosuppressive properties of human amnion- and chorion-derived mesenchymal stem cells. PLoS One. 2014; 9: e88319.

72. Im KI, Park MJ, Kim N, Lim JY, Park HS, Lee SH, Nam YS, Lee ES, Lee JH, Cho ML, Cho SG. Induction of mixed chimerism using combinatory cell-based immune modulation with mesenchymal stem cells (MSCS) and regulatory T cells (TREGS) for solid organ transplantaion. Haematologica. 2014; 99: 160.

73. Im K, Kim N, Lim J, Nam Y, Lee E, Chae E, Kim E, Cho S. Induction of mixed chimerism using combinatory cell-based immune modulation with MSCS and Tregs in early post-transplant period. Cytotherapy. 2014; 16: S36.

74. Sadeghi B, Erkers T, Heshmati Y, Magalhaes I, Nava S, Mollden P, Walfridsson J, Kaipe H, Ringden O. Experimental and clinical studies using decidual stromal cells from fetal membrane layers for immune modulation and graft-versus-host disease. Bone Marrow Transplant. 2014; 49: S128.

75. Roux C, Saviane G, Dhib G, Pini J, Rohrlich PS, Belaid N, Wakkach A, Blin C, Rouleau M. Immunosuppression by mesenchymal stromal cells derived from human induced pluripotent stem cells : evaluation in an aGVHD model. Blood. 2014; 124: 1097.

76. Robles J, Liu Y, Cao J, Xiang Z, Manio M, Choi Y, Xu J, Tsia K, Tang E, Chan G. Immunosuppressive mechanisms of human mesenchymal stem cells in graft vesus host disease murine models. Transplantation. 2014; 98: 333.

77. Auletta JJ, Eid SK, Wuttisarnwattana P, Keller MD, Silva I, Wilson DL, Cooke KR. Defining the biodistribution and immunomodulatory mechanisms of human mesenchymal stromal cells following allogeneic bmt. Bone Marrow Transplant. 2014; 49: S314-S315.

78. Heider A, Müller AM, Hilger N, Niederwieser D, Cross M, Alt R, Hansen M, Hoffmann A, Fricke S. Umbilical cord derived mesenchymal stromal cells are attractive candidates for a novel immunosuppressive therapy approach against graft-versus-host-disease after umbilical cord blood transplantation. Oncology Research and Treatment. 2014; 37: 109-110.

79. Barrett A, Amarnath S, Salem B, Laurence A, Eckhaus M, Melenhorst JJ, Rose JJ, Sabatino M, Stroncek D, Fowler DH. Bone marrow derived mesenchymal stromal cells (BMSC) are efficient modulators of xenogeneic graft versus host disease. Bone Marrow Transplant. 2014; 49: S314.

80. Wen F, Zhang HJ, Yin W, et al. SCA1+ mesenchymal stromal cells inhibit graft-versushost disease in mice after bone marrow transplantation. Haematologica. 2015; 100: 618.

81. Wang J, Yu M, Chen S, Zhao J, Zhang Y, Lin X, Zhe N, Fang Q. HO-1-transduced mesenchymal stem cells attenuate AGVHD by inhibiting HIF-1α /ROR-γt and then down-regulate proportion of TH17/Treg. Blood. 2015; 126: 5421.

82. Sánchez-Abarca LI, Hernández-Galilea E, Lorenzo R, Herrero C, Velasco A, Carrancio S, Caballero-Velázquez T, Rodríguez-Barbosa JI, Parrilla M, Del Cañizo C, San Miguel J, Aijón J, Pérez-Simón JA. Human Bone marrow stromal cells differentiate into corneal tissue and prevent ocular graft-versus-host disease in mice. Cell Transplant. 2015; 24: 2423-2433.

83. Roux C, Saviane G, Dhib G, Pini J, Rohrlich PS, Belaid N, Wakkach A, Blin C, Rouleau M. Immunosuppression by mesenchymal stromal cells derived from human induced pluripotent stem cells: evaluation in an agvhd model. Haematologica. 2015; 100: 184-185.

84. Ringdén O, Solders M, Erkers T, Nava S, Molldén P, Khoein B, Baygan A, Aronsson-Kurttila W, Mohmand S, Remberger M, Westgren M. Placenta-derived decidual stromal cells for graft-versus-host disease, hemorrhaging, and toxicity after allogeneic hematopoietic stem cell transplantation. Bone Marrow Transplant. 2015; 50: S315-S316.

85. Nagamura-Inoue T, Mori Y, Takahashi A, Shimazu T, Tsunoda H, Tojo A. Simple, efficient, and safe processing of frozen-thawed umbilical cord-derived mesenchymal stromal cells applied for the treatment of gvhd. Haematologica. 2015; 100: 275-276.

86. Jiang Q, Huang H, Zhou Y, Zhang Q, Sun X, Zhang M, Ye Y, Zheng Z, Ye K, Jerry KM, Sun J. Target transplantation of mesenchymal stem cells into the murine femur to support hematopoiesis by MagIC-TT: both in vivo and in vitro studies. Blood. 2015; 126: 5409.

87. Gu J, Ling L. Human gingival tissue-derived mesenchymal stem cells reduce murine acute GVHD via killing of effector CD8+ cells through CD39/adenosine A1R and A2BR pathways. Am J Transplant. 2015; 15.

88. Amarnath S, Foley JE, Farthing DE, Gress RE, Laurence A, Eckhaus MA, Métais JY, Rose JJ, Hakim FT, Felizardo TC, et al. Bone marrow-derived mesenchymal stromal cells harness purinergenic signaling to tolerize human Th1 cells in vivo. Stem Cells. 2015; 33: 1200-1212.
