# Supplementary material for: Mesenchymal stem cells provide prophylaxis against acute graft-versus-host disease following allogeneic hematopoietic stem cell transplantation: A meta-analysis of animal models
Source: Oncotarget. 2016 Aug 12;7(38):61764–74. doi: 10.18632/oncotarget.11238 (PMC5308689; doi:10.18632/oncotarget.11238)
Supplement: Supplementary file 3 [file oncotarget-07-61764-s003.docx]

**Table S2. Characteristics of the included studies**

| **Author** | **Allo-HSCT model** | | | | | **MSCs intervention** | | | | | **Control** | |
| --- | --- | --- | --- | --- | --- | --- | --- | --- | --- | --- | --- | --- |
|  | **Recipients** | **Sex** | **Age** | **Donors** | **graft** | **Species** | **Tissue origin** | **Dose (×10^6^)** | **Administration time** | **No. of**  **MSC-treated** | **Regimen** | **No. of**  **controls** |
| Deng 2003 ^[1]^ | SD rat | NA | 4-6W | F344 rat | BM+SPC | SD rat | BM | 1 | d0 | 10 | NA | 10 |
| Chung 2004 ^[2]^ | Balb/c mice | F | 4-6W | C3H/He mice | BM+SPC | C3H/He mice | BM | 0.1 | d0 | 11 | NA | 11 |
| Sudres 2006 ^[3]^ | Balb/c mice | F | 8-12W | C57BL/6 mice | BM+CD3^+^ T | C57BL/6 mice | BM | 0.5 or 3 or 4 | d0 | 25 | NA | 15 |
| Yanez 2006 ^[4]^ | CB6F1 mice | NA | 10-12W | C57BL/6 mice | BM+SPC | CB6F1 mice | Adipose tissue | 0.05 | d0 & d7 & d14 | 10 | NA | 10 |
| Yanez 2006 ^[4]^ | CB6F1 mice | NA | 10-12W | C57BL/6 mice | BM+SPC | CB6F1 mice | Adipose tissue | 0.05 | d14 & d21 & d28 | 10 | NA | 10 |
| Li 2006 ^[5]^ | C57BL/6 mice | NA | 8-12W | Balb/c mice | TCD BM+SPC | Balb/c mice | BM | 5 | d0 | 7 | NA | 7 |
| Tisato 2007 ^[6]^ | NSG mice | NA | 6-10W | Human | PBMC | Human | UCB | 3 | d0 & d7 & d14 & d21 | 5 | PBS | 5 |
| Tisato 2007 ^[6]^ | NSG mice | NA | 6-10W | Human | PBMC | Human | UCB | 3 | d0 | 5 | PBS | 5 |
| Min 2007 ^[7]^ | F1 mice | F | 8-12W | C57BL/6 mice | BM+ SPC | C57BL/6 mice | BM | 1 or 2 | d1 | 20 | Diluent | 10 |
| Li 2008 ^[8]^ | Balb/c mice | F | NA | C57BL/6 mice | SPC | C57BL/6 mice | BM | 0.02 or 0.2 or 1 or 2 | d0 or d3 | 20 | NA | 20 |
| Ren 2008 ^[9]^ | F1 mice | M | 8W | C57BL/6 mice | BM+SPC | C57BL/6 mice | BM | 0.5 | d3 & d7 | 5 | NA | 5 |
| Tian 2008 ^[10]^ | Wistar rats | NA | NA | Fischer 344 rats | BM+SPC | Fischer 344 rats | BM | 2 | d0 | 10 | PBS | 10 |
| Polchert 2008 ^[11]^ | C57BL/6 mice | F | 10-12W | Balb/c mice | BM+SPC | mice | BM | 0.1 or 0.5 | d0 or d2 | 30 | NA | 30 |
| Badillo 2008 ^[12]^ | B6×BALB/c F1 mice | NA | 8-12W | C57BL/6 mice | BM+SPC | C57BL/6 mice | BM | 0.05 or 0.15 or 1 | d0 or d2 or (d0 & d7 & d14) |  | NA |  |
| Lu 2009 ^[13]^ | F1 mice | NA |  | C57BL/6 mice | BM+SPC | C57BL/6 mice | BM | 2 | d0 | 17 | NA | 17 |
| Christensen 2010 ^[14]^ | Balb/c mice | F | 6-8W | C57BL/6 mice | BM+SPC | C57BL/6 mice | BM | 0.4 | d1 | 18 | Medium | 28 |
| Prigozhina 2010 ^[15]^ | CB6F1 mice | NA | 2M | C57BL/6 mice | BM | C57BL/6 or CB6F1 mice | BM | 0.05 or 0.5 | d0 & d7 & d14 | 10 | NA | 10 |
| Zhang 2010 ^[16]^ | CB6F1 mice | M | 8-12W | C57BL/6 mice | BM | Human | UCB | 1 | d0 | 20 | PBS | 20 |
| Joo 2010 ^[17]^ | Balb/c mice | F | 8-12W | C3H/he mice | BM+SPC | C3H/he mice | BM | 0.5 or 1 or 2 | d0 | 8 | NA | 8 |
| Hao 2011 ^[18]^ | (B6×BALB/c) F1 mice | M | 8-10W | C57BL/6 mice | BM+SPC | Human | UCB | 10 | d0 | 10 | NA | 10 |
| Guo 2011 ^[19]^ | DBA/2 mice | M | 10-12W | C57BL/6 mice | BM+SPC | Human | UC | 1 | d0 | 10 | NA | 10 |
| Zhang 2011 ^[20]^ | CB6F1 mice | F | 8-10W | C57BL/6 mice | BM+SPC | Human | UCB | 1 | d0 | 20 | NA | 20 |
| Gregoire-Gauthier 2012 ^[21]^ | NSG mice | NA | 6-8W | Human | PBMC | Human | UCB | 1 | d0 | 16 | PBS | 16 |
| Kitazawa 2012 ^[22]^ | (Lewis × DA) F1 rat | M | 4-6W | Lewis rat | LN cells | Lewis rat | BM | 10 | d1 & d6 | 10 | NA | 7 |
| Zinocker 2012 ^[23]^ | BN⁄ RijHsd rats | M&  F | 9-12W | PVG.7B rats | TCD BM+LN cells | PVG.7B rats | BM | 1-2 | d0 & d7 & d14 | 8 | PBS | 6 |
| Zinocker 2012 ^[23]^ | PVG.R23 rats | M&  F | 9-12W | PVG.1U rats | TCD BM+LN cells | PVG.1U rats | BM | 1-2 | d0 & d7 & d14 | 10 | PBS | 10 |
| Zinocker 2012 ^[23]^ | PVG.R23 rats | M&  F | 9-12W | PVG.1U rats | TCD BM+LN cells | PVG.7B rats | BM | 1 | d0 & d7 & d16 | 6 | PBS | 5 |
| Kikuchi-Taura 2012 ^[24]^ | B6C3F1 mice | F | 8-12W | BDF1 mice | BM+SPC | Human | UC | 1 | d0 & d7 | 5 | Medium | 5 |
| Chen 2012 ^[25]^ | Balb/c mice | NA | 6-8W | C57BL/6 mice | BM+SPC | C57BL/6 mice | BM | 0.2 | d1 & d7 | 10 | NA | 10 |
| Bruck 2013 ^[26]^ | NSG mice | NA | NA | Human | PBMC | Human | BM | 2 or 3 | d0 or (d0 & d7 & d14 & d21) | 53 | NA | 61 |
| Wang 2013 ^[27]^ | Balb/c mice | F | NA | C57BL/6 mice | BM+SPC | Balb/c mice | BM | 2 | d0 | 10 | NA | 10 |
| Tobin 2013 ^[28]^ | NSG mice | NA | NA | Human | PBMC | Human | BM | 1 | d7 | 7 | PBS | 7 |
| Yoo 2013 ^[29]^ | Balb/c mice | M | 10 W | C57BL/6 mice | BM+SPC | C3H/HeN mice | BM | 0.5 | d1 & d3 | 27 | Vehicle | 19 |
| Yoo 2013 ^[29]^ | Balb/c mice | M | 10 W | C57BL/6 mice | BM+SPC | Human | BM | 0.5 | d1 & d3 | 49 | Vehicle | 30 |
| Oviedo 2013 ^[30]^ | B6D2F1 mice | NA | 10-12 W | C57BL/6 mice | BM+SPC | B6D2F1 mice | Adipose tissue | 0.2-0.25 | d0 & d7 & d14 | 20 | NA | 20 |
| Nevruz 2013 ^[31]^ | Wistar rat | F | 10-12 W | Sprague Dawley rat | BM+SPC | Sprague Dawley rat | BM | 0.4 | d1 | 7 | NA | 7 |
| Jang 2013 ^[32]^ | Balb/c mice | F | 6-8 W | C57BL/6 mice | BM+SPC | Human | Placenta | 0.5 or 1 | d0 | 27 | NA | 12 |
| Lim 2014 ^[33]^ | Balb/c mice | F | 8-10 W | C57BL/6 mice | BM+SPC | Balb/c mice | BM | 1 | d0 & d4 | 6 | PBS | 6 |
| Xishan 2014 ^[34]^ | Balb/c mice | M | 6-8 W | C57BL/6 mice | BM | Balb/c mice | BM | 0.1 or 1 | d0 | 10 | NA | 10 |
| Li 2014 ^[35]^ | Balb/c mice | F | 6-8 W | C57BL/6 mice | UCB | C57BL/6 mice | BM | 20 | d0 | 10 | NA | 10 |
| Li 2014 ^[36]^ | C57BL/6 mice | M | NA | Balb/c mice | BM+SPC | mice | BM | 0.1 or 0.5 | d0 | 24 | NA | 12 |
| Jang 2014 ^[37]^ | NSG mice | M | 9W | Human | PBMC | Human | UCB | 0.5 | d0 or (d0 & d3 & d6) or (d0 & d7 & d14) | 30 | Medium | 10 |
| Im 2014 ^[38]^ | Balb/c mice | F | 8 W | C57BL/6 mice | BM+SPC | Human | Adipose tissue | 2 | d1 & d10 | 6 | NA | 6 |
| Gao 2014 ^[39]^ | Balb/c mice | F | NA | C57BL/6 mice | BM+SPC | C57BL/6 mice | Dermal | 2 | d0 | 24 | NA | 24 |
| Girdlestone 2015 ^[40]^ | Balb/c mice | M&  F | 8-15 W | Human | PBMC | Human | UC | 0.5 or 2 | d8 | 12 | PBS | 8 |
| Yang 2015 ^[41]^ | Balb/c mice | F | 6-8 W | C57BL/6 mice | BM+SPC | Mice | BM | 0.2 | d0 | 20 | PBS | 20 |
| Yang 2015 ^[41]^ | CB6F1 mice | F | 6-8 W | C57BL/6 mice | BM+SPC | C57BL/6 mice | BM | 0.2 | d0 | 35 | PBS | 35 |
| Wen 2015 ^[42]^ | Balb/c mice | M&  F | 8 W | C57BL/6 mice | BM+SPC | C57BL/6 mice | BM | 0.08 | d0 | 22 | NA | 12 |
| Song 2015 ^[43]^ | Balb/c mice | NA | 6-8 W | C57BL/6 mice | BM | C57BL/6 mice | BM | 0.5 | d0 | 10 | NA | 10 |
| Sadeghi 2015 ^[44]^ | Balb/c mice | F | 10-12 W | C57BL/6 mice | BM+SPC | Human | Decidua | 0.1 | d0 or d3 or d5 or d7 or (d3 & d5) | 32 | NA | 8 |
| Robles 2015 ^[45]^ | Balb/c mice | M | 6-8 W | C57BL/6 mice | TCD BM+CD4^+^T | Human | BM | 1 | d0 & d3 & d6 | 12 | NA | 20 |
| Auletta 2015 ^[46]^ | F1 mice | F | 8-12W | C57BL/6 mice | BM+SPC | Human | BM | 1 | d0 & d4 | 30 | NA | 30 |
| Lee 2015 ^[47]^ | Balb/c mice | NA | 8-10W | C57BL/6 mice | BM+SPC | C57BL/6 mice | BM | 1 | d0 & d4 | 10 | PBS | 10 |
| Hinden 2015 ^[48]^ | F1 mice | F | 8-11W | C57BL/6 mice | BM+SPC | C57BL/6 mice | BM | 1 | d0 | 10 | NA | 10 |
| Kim 2015 ^[49]^ | Balb/c mice | F | 8-10W | C57BL/6 mice | BM+SPC | Human | Adipose tissue | 1 | d1 | 12 | PBS | 12 |
| Luz-Crawford 2016 ^[50]^ | NSG mice | NA | 8-10W | human | PBMC | Human | BM or menstrual blood | 1 | d0 | 20 | NA | 10 |

Abbreviations: allo-HSCT: allogeneic hematopoietic stem cell transplantation, MSCs: mesenchymal stem cells, BM: bone marrow, LN: lymph node, SPC: splenocytes, UCB: umbilical cord blood, UC: umbilical cord, M: male, F: female, W: week, M: month, TCD: T cell-depleted, NA: not available, PBS: phosphate-buffered saline, PBMC: peripheral blood mononuclear cells.

**REFERENCES**

1. Deng Y, Guo X, Yuan Q, Li S. Efficiency of adenoviral vector mediated CTLA4Ig gene delivery into mesenchymal stem cells. Chin Med J (Engl). 2003; 116: 1649-1654.

2. Chung NG, Jeong DC, Park SJ, Choi BO, Cho B, Kim HK, Chun CS, Won JH, Han CW. Cotransplantation of marrow stromal cells may prevent lethal graft-versus-host disease in major histocompatibility complex mismatched murine hematopoietic stem cell transplantation. Int J Hematol. 2004; 80: 370-376.

3. Sudres M, Norol F, Trenado A, Grégoire S, Charlotte F, Levacher B, Lataillade JJ, Bourin P, Holy X, Vernant JP, Klatzmann D, Cohen JL. Bone marrow mesenchymal stem cells suppress lymphocyte proliferation in vitro but fail to prevent graft-versus-host disease in mice. J Immunol. 2006; 176: 7761-7767.

4. Yañez R, Lamana ML, García-Castro J, Colmenero I, Ramírez M, Bueren JA. Adipose tissue-derived mesenchymal stem cells have in vivo immunosuppressive properties applicable for the control of the graft-versus-host disease. Stem Cells. 2006; 24: 2582-2591.

5. Li A, Zhang Q, Jiang J, Yuan G, Feng Y, Hao J, Li C, Gao X, Wang G, Xie S. Co-transplantation of bone marrow stromal cells transduced with IL-7 gene enhances immune reconstitution after allogeneic bone marrow transplantation in mice. Gene Ther. 2006; 13: 1178-1187.

6. Tisato V, Naresh K, Girdlestone J, Navarrete C, Dazzi F. Mesenchymal stem cells of cord blood origin are effective at preventing but not treating graft-versus-host disease. Leukemia. 2007; 21: 1992-1999.

7. Min CK, Kim BG, Park G, Cho B, Oh IH. IL-10-transduced bone marrow mesenchymal stem cells can attenuate the severity of acute graft-versus-host disease after experimental allogeneic stem cell transplantation. Bone Marrow Transplant. 2007; 39: 637-645.

8. Li H, Guo Z, Jiang X, Zhu H, Li X, Mao N. Mesenchymal stem cells alter migratory property of T and dendritic cells to delay the development of murine lethal acute graft-versus-host disease. Stem Cells. 2008; 26: 2531-2541.

9. Ren G, Zhang L, Zhao X, Xu G, Zhang Y, Roberts AI, Zhao RC, Shi Y. Mesenchymal stem cell-mediated immunosuppression occurs via concerted action of chemokines and nitric oxide. Cell Stem Cell. 2008; 2: 141-150.

10. Tian Y, Deng YB, Huang YJ, Wang Y. Bone marrow-derived mesenchymal stem cells decrease acute graft-versus-host disease after allogeneic hematopoietic stem cells transplantation. Immunol Invest. 2008; 37: 29-42.

11. Polchert D, Sobinsky J, Douglas G, Kidd M, Moadsiri A, Reina E, Genrich K, Mehrotra S, Setty S, Smith B, Bartholomew A. IFN-gamma activation of mesenchymal stem cells for treatment and prevention of graft versus host disease. Eur J Immunol. 2008; 38: 1745-1755.

12. Badillo AT, Peranteau WH, Heaton TE, Quinn C, Flake AW. Murine bone marrow derived stromal progenitor cells fail to prevent or treat acute graft-versus-host disease. Br J Haematol. 2008; 141: 224-234.

13. Lu X, Liu T, Gu L, Huang C, Zhu H, Meng W, Xi Y, Li S, Liu Y. Immunomodulatory effects of mesenchymal stem cells involved in favoring type 2 T cell subsets. Transpl Immunol. 2009; 22: 55-61.

14. Christensen ME, Turner BE, Sinfield LJ, Kollar K, Cullup H, Waterhouse NJ, Hart DN, Atkinson K, Rice AM. Mesenchymal stromal cells transiently alter the inflammatory milieu post-transplant to delay graft-versus-host disease. Haematologica. 2010; 95: 2102-2110.

15. Prigozhina TB, Khitrin S, Elkin G, Eizik O, Morecki S, Slavin S. Mesenchymal stromal cells lose their immunosuppressive potential after allotransplantation. Exp Hematol. 2008; 36: 1370-1376.

16. Zhang C, Chen XH, Zhang X, Gao L, Kong PY, Peng XG, Liang X, Gao L, Wang QY. Human umbilical cord blood-derived stromal cells: A new resource in hematopoietic reconstitution in mouse haploidentical transplantation. Transplant Proc. 2010; 42: 3739-3744.

17. Joo SY, Cho KA, Jung YJ, Kim HS, Park SY, Choi YB, Hong KM, Woo SY, Seoh JY, Cho SJ, Ryu KH. Mesenchymal stromal cells inhibit graft-versus-host disease of mice in a dose-dependent manner. Cytotherapy. 2010; 12: 361-370.

18. Hao L, Gao L, Chen XH, Zou ZM, Zhang X, Kong PY, Zhang C, Peng XG, Sun AH, Wang QY. Human umbilical cord blood-derived stromal cells prevent graft-versus-host disease in mice following haplo-identical stem cell transplantation. Cytotherapy. 2011; 13: 83-91.

19. Guo J, Yang J, Cao G, Fan H, Guo C, Ma YE, Qian Y, Chen L, Li X, Chang C. Xenogeneic immunosuppression of human umbilical cord mesenchymal stem cells in a major histocompatibility complex-mismatched allogeneic acute graft-versus-host disease murine model. Eur J Haematol. 2011; 87: 235-243.

20. Zhang C, Chen XH, Zhang X, Gao L, Kong PY, Peng XG, Liang X, Gao L, Gong Y, Wang QY. Human umbilical cord blood-derived stromal cells, a new resource in the suppression of acute graft-versus-host disease in haploidentical stem cell transplantation in sublethally irradiated mice. J Biol Chem. 2011; 286: 13723-13732.

21. Gregoire-Gauthier J, Selleri S, Fontaine F, Dieng MM, Patey N, Despars G, Beauséjour CM, Haddad E. Therapeutic efficacy of cord blood-derived mesenchymal stromal cells for the prevention of acute graft-versus-host disease in a xenogenic mouse model. Stem Cells Dev. 2012; 21: 1616-1626.

22. Kitazawa Y, Li XK, Xie L, Zhu P, Kimura H, Takahara S. Bone marrow-derived conventional, but not cloned, mesenchymal stem cells suppress lymphocyte proliferation and prevent graft-versus-host disease in rats. Cell Transplant. 2012; 21: 581-590.

23. Zinöcker S, Wang MY, Rolstad B, Vaage JT. Mesenchymal stromal cells fail to alleviate experimental graft-versus-host disease in rats transplanted with major histocompatibility complex-mismatched bone marrow. Scand J Immunol. 2012; 76: 464-470.

24. Kikuchi-Taura A, Taguchi A, Kanda T, Inoue T, Kasahara Y, Hirose H, Sato I, Matsuyama T, Nakagomi T, Yamahara K, Stern D, Ogawa H, Soma T. Human umbilical cord provides a significant source of unexpanded mesenchymal stromal cells. Cytotherapy. 2012; 14: 441-450.

25. Chen W, Li M, Li Z, Yan Z, Cheng H, Pan B, Cao J, Chen C, Zeng L, Xu K. CXCR4-transduced mesenchymal stem cells protect mice against graft-versus-host disease. Immunol Lett. 2012; 143: 161-169.

26. Bruck F, Belle L, Lechanteur C, de Leval L, Hannon M, Dubois S, Castermans E, Humblet-Baron S, Rahmouni S, Beguin Y, Briquet A, Baron F. Impact of bone marrow-derived mesenchymal stromal cells on experimental xenogeneic graft-versus-host disease. Cytotherapy. 2013; 15: 267-279.

27. Wang YC, Wang SH, Wei YN, Du DW, Xu H, Gao CC, Zheng MH, Xie J, Li JC, Dong GY, Li L, Xiao Y, Han H. Notch-RBP-J signaling is required by bone marrow stromal cells for the treatment of acute graft versus host disease. Stem Cell Res. 2013; 11: 721-735.

28. Tobin LM, Healy ME, English K, Mahon BP. Human mesenchymal stem cells suppress donor CD4(+) T cell proliferation and reduce pathology in a humanized mouse model of acute graft-versus-host disease. Clin Exp Immunol. 2013; 172: 333-348.

29. Yoo HS, Yi T, Cho YK, Kim WC, Song SU, Jeon MS. Mesenchymal stem cell lines isolated by different isolation methods show variations in the regulation of graft-versus-host disease. Immune Netw. 2013; 13: 133-140.

30. Oviedo A, Yañez R, Colmenero I, Aldea M, Rubio A, Bueren JA, Lamana ML. Reduced efficacy of mesenchymal stromal cells in preventing graft-versus-host disease in an in vivo model of haploidentical bone marrow transplant with leukemia. Cell Transplant. 2013; 22: 1381-1394.

31. Nevruz O, Avcu F, Ural AU, Pekel A, Dirican B, Safalı M, Akdağ E, Beyzadeoğlu M, Ide T, Sengül A. Immunosuppressive effects of multipotent mesenchymal stromal cells on graft-versus-host disease in rats following allogeneic bone marrow transplantation. Turk J Haematol. 2013; 30: 256-262.

32. Jang MJ, Kim HS, Lee HG, Kim GJ, Jeon HG, Shin HS, Chang SK, Hur GH, Chong SY, Oh D, Chung HM. Placenta-derived mesenchymal stem cells have an immunomodulatory effect that can control acute graft-versus-host disease in mice. Acta Haematol. 2013; 129: 197-206.

33. Lim JY, Park MJ, Im KI, Kim N, Jeon EJ, Kim EJ, Cho ML, Cho SG. Combination cell therapy using mesenchymal stem cells and regulatory T-cells provides a synergistic immunomodulatory effect associated with reciprocal regulation of TH1/TH2 and th17/treg cells in a murine acute graft-versus-host disease model. Cell Transplant. 2014; 23: 703-714.

34. Xishan Z, Haojun Y, Baoxin H, Xinna Z, Ni J, Hongmei Z, Xiaoli W, Jun R. Mouse Flk-1+Sca-1- mesenchymal stem cells: functional plasticity in vitro and immunoregulation in vivo. Transplantation. 2014; 97: 509-517.

35. Li ZY, Wang CQ, Lu G, Pan XY, Xu KL. Effects of bone marrow mesenchymal stem cells on hematopoietic recovery and acute graft-versus-host disease in murine allogeneic umbilical cord blood transplantation model. Cell Biochem Biophys. 2014; 70: 115-122.

36. Li H, Jiang Y, Jiang X, Guo X, Ning H, Li Y, Liao L, Yao H, Wang X, Liu Y, Zhang Y, Chen H, Mao N. CCR7 guides migration of mesenchymal stem cell to secondary lymphoid organs: a novel approach to separate GvHD from GvL effect. Stem Cells. 2014; 32: 1890-1903.

37. Jang YK, Kim M, Lee YH, Oh W, Yang YS, Choi SJ. Optimization of the therapeutic efficacy of human umbilical cord blood-mesenchymal stromal cells in an NSG mouse xenograft model of graft-versus-host disease. Cytotherapy. 2014; 16: 298-308.

38. Im KI, Park MJ, Kim N, Lim JY, Park HS, Lee SH, Nam YS, Lee ES, Lee JH, Cho ML, Cho SG. Induction of mixed chimerism using combinatory cell-based immune modulation with mesenchymal stem cells and regulatory T cells for solid-organ transplant tolerance. Stem Cells Dev. 2014; 23: 2364-2376.

39. Gao L, Liu F, Tan L, Liu T, Chen Z, Shi C. The immunosuppressive properties of non-cultured dermal-derived mesenchymal stromal cells and the control of graft-versus-host disease. Biomaterials. 2014; 35: 3582-3588.

40. Girdlestone J, Pido-Lopez J, Srivastava S, Chai J, Leaver N, Galleu A, Lombardi G, Navarrete CV. Enhancement of the immunoregulatory potency of mesenchymal stromal cells by treatment with immunosuppressive drugs. Cytotherapy. 2015; 17: 1188-1199.

41. Yang D, Wang LP, Zhou H, Cheng H, Bao XC, Xu S, Zhang WP, Wang JM. Inducible Costimulator Gene-transduced Bone marrow-derived mesenchymal stem cells attenuate the severity of acute graft-versus-host disease in mouse models. Cell Transplant. 2015; 24: 1717-1731.

42. Wen F, Zhang HJ, Chen Y, Yue Q, Liu Z, Zhang Q, An N, Chen X, Li N, Xin J, et al. Sca1(+) mesenchymal stromal cells inhibit graft-versus-host disease in mice after bone marrow transplantation. Int Immunopharmacol. 2015; 26: 50-57.

43. Song N, Gao L, Qiu H, Huang C, Cheng H, Zhou H, Lv S, Chen L, Wang J. Mouse bone marrow-derived mesenchymal stem cells inhibit leukemia/lymphoma cell proliferation in vitro and in a mouse model of allogeneic bone marrow transplant. Int J Mol Med. 2015; 36: 139-149.

44. Sadeghi B, Heshmati Y, Khoein B, Kaipe H, Uzunel M, Walfridsson J, Ringdén O. Xeno-immunosuppressive properties of human decidual stromal cells in mouse models of alloreactivity in vitro and in vivo. Cytotherapy. 2015; 17: 1732-1745.

45. Robles JD, Liu YP, Cao J, Xiang Z, Cai Y, Manio M, Tang EH, Chan GC. Immunosuppressive mechanisms of human bone marrow derived mesenchymal stromal cells in BALB/c host graft versus host disease murine models. Exp Hematol Oncol. 2015; 4: 13.

46. Auletta JJ, Eid SK, Wuttisarnwattana P, Silva I, Metheny L, Keller MD, Guardia-Wolff R, Liu C, Wang F, Bowen T, et al. Human mesenchymal stromal cells attenuate graft-versus-host disease and maintain graft-versus-leukemia activity following experimental allogeneic bone marrow transplantation. Stem Cells. 2015; 33: 601-614.

47. Lee ES, Lim JY, Im KI, Kim N, Nam YS, Jeon YW, Cho SG. Adoptive transfer of Treg cells combined with mesenchymal stem cells facilitates repopulation of endogenous Treg cells in a murine acute GVHD model. PLOS ONE. 2015; 10: e0138846.

48. Hinden L, Shainer R, Almogi-Hazan O, Or R. Ex vivo induced regulatory human/murine mesenchymal stem cells as immune modulators. Stem Cells. 2015; 33: 2256-2267.

49. Kim KW, Moon SJ, Park MJ, Kim BM, Kim EK, Lee SH, Lee EJ, Chung BH, Yang CW, Cho ML. Optimization of adipose tissue-derived mesenchymal stem cells by rapamycin in a murine model of acute graft-versus-host disease. Stem Cell Res Ther. 2015; 6: 202.

50. Luz-Crawford P, Torres MJ, Noël D, Fernandez A, Toupet K, Alcayaga-Miranda F, Tejedor G, Jorgensen C, Illanes SE, Figueroa FE, Djouad F, Khoury M. The immunosuppressive signature of menstrual blood mesenchymal stem cells entails opposite effects on experimental arthritis and graft versus host diseases. Stem Cells. 2016; 34: 456-469.
